# Superior Capsule Reconstruction Graft Selection: The Influence of Biological Properties of Grafts on Healing and Re-Tearing

**DOI:** 10.3390/bioengineering12090942

**Published:** 2025-08-31

**Authors:** Mingde Cao, Mingguang Bi, Shuai Yuan, Yuhao Wu, Patrick Shu-Hang Yung, Michael Tim-Yun Ong

**Affiliations:** 1 Department of Orthopaedics and Traumatology, Faculty of Medicine, Shatin, Hong Kong SAR, China; mingdecao@link.cuhk.edu.hk (M.C.);; 2 Center for Neuromusculoskeletal Restorative Medicine (CNRM), The Chinese University of Hong Kong, Hong Kong SAR, China; 3 Department of Joint and Sports Medicine, The Affiliated Lihuili Hospital, Ningbo University, Ningbo 315211, China; bmg_vip@163.com; 4 Department of Sport Medicine, The Third Affiliated Hospital, Southern Medical University, Guangzhou 510515, China

**Keywords:** superior capsule reconstruction, graft healing, autograft

## Abstract

Arthroscopic Superior Capsular Reconstruction has emerged as a promising surgical intervention for irreparable massive rotator cuff tears, aiming to restore glenohumeral joint stability and improve patient outcomes. A critical determinant of ASCR success is the selection of an appropriate graft material. This review explores the spectrum of grafts utilized in ASCR, including autografts, allografts, xenografts, and synthetic materials. The primary focus is on how the inherent biological properties of these grafts—such as cellularity, vascularity, immunogenicity, and extracellular matrix composition—profoundly influence the processes of graft healing, integration into host tissues, and ultimately, the rates of re-tearing. Autografts, particularly fascia lata, often demonstrate superior biological incorporation due to their viable cells and non-immunogenic nature, leading to high healing rates. Allografts, while offering advantages like reduced donor site morbidity, present biological challenges related to decellularization processes and slower remodeling, resulting in more variable healing outcomes. Xenografts face significant immunological hurdles, often leading to rejection and poor integration. Synthetic grafts provide an off-the-shelf option but interact with host tissue primarily as a scaffold, without true biological integration. Understanding the nuanced biological characteristics of each graft type is paramount for surgeons aiming to optimize healing environments and minimize re-tear rates, thereby enhancing the long-term efficacy of ASCR.

## 1. Introduction

Irreparable massive rotator cuff tears present a significant clinical challenge, often leading to debilitating shoulder pain, loss of function, and diminished quality of life [1]. Traditional treatments, including debridement, partial repair, or tendon transfers, may yield suboptimal results in this patient population [2]. Arthroscopic Superior Capsular Reconstruction (ASCR) has gained prominence as a viable surgical technique designed to address the biomechanical deficits associated with these extensive tears [3]. The primary goal of ASCR is to restore the superior stability of the glenohumeral joint by reconstructing the deficient superior capsule, thereby recentering the humeral head within the glenoid fossa, reducing pain, and improving shoulder function [4].

The success of ASCR is multifactorial, but the choice of graft material stands out as a pivotal element. The graft must not only provide immediate mechanical stability but also integrate biologically with the host tissues to ensure durable, long-term function [5]. A variety of graft materials have been employed, ranging from the patient’s own tissues (autografts) to donor tissues (allografts), animal-derived tissues (xenografts), and man-made materials (synthetic grafts) [6,7]. Each of these options possesses distinct biological properties that significantly impact their handling, incorporation, healing cascade, and susceptibility to failure through re-tearing [8,9].

This narrative review explores the various graft types utilized in ASCR, focusing on how their inherent biological properties impact the intricate processes of graft healing, integration, and the risk of re-tearing. To gather relevant evidence, a systematic search was conducted in the PubMed, Cochrane Library, and Embase databases for full-text, English-language journal articles published between 1 August 2015, and 1 August 2025. The search employed the following keywords: “superior capsular reconstruction”, “superior capsule reconstruction”, “ASCR”, and “graft”.

## 2. The Biological Imperative in Graft Healing for ASCR

Successful ASCR hinges on the biological integration of the chosen graft material [10]. Graft healing in the shoulder, a dynamic and mechanically demanding joint, involves a complex cascade of biological events. These include revascularization (the formation of new blood vessels into the graft), cellular repopulation (migration of host cells, such as fibroblasts and tenocytes, into the graft scaffold), and extracellular matrix (ECM) remodeling (the gradual replacement and reorganization of the graft’s matrix to resemble native capsular tissue) [11].

The ideal graft should be biocompatible, minimizing inflammatory or immune responses that could impede healing or lead to graft rejection [12]. It should possess an architecture that supports cellular infiltration and vascular ingrowth. Furthermore, its mechanical properties must withstand the physiological loads experienced by the shoulder during the healing phase and beyond [13]. The biological properties of the graft material itself—whether it contains viable cells, its ECM composition, immunogenicity, and its degradation profile—are central to these processes. A graft that facilitates a robust and timely biological response is more likely to achieve durable integration and restore long-term joint stability, whereas a graft with unfavorable biological characteristics may lead to incomplete healing, fibrous encapsulation rather than true integration, or eventual mechanical failure and re-tearing [14,15,16].

## 3. Autografts: Leveraging Native Biology for Enhanced Healing

Autografts are often considered the gold standard in reconstructive surgery due to their inherent biological advantages. They are non-immunogenic, eliminating the risk of immune rejection, and may contain viable cells that can actively participate in the healing and remodeling process [16].

### 3.1. Fascia Lata Autograft

**Figure 1:**
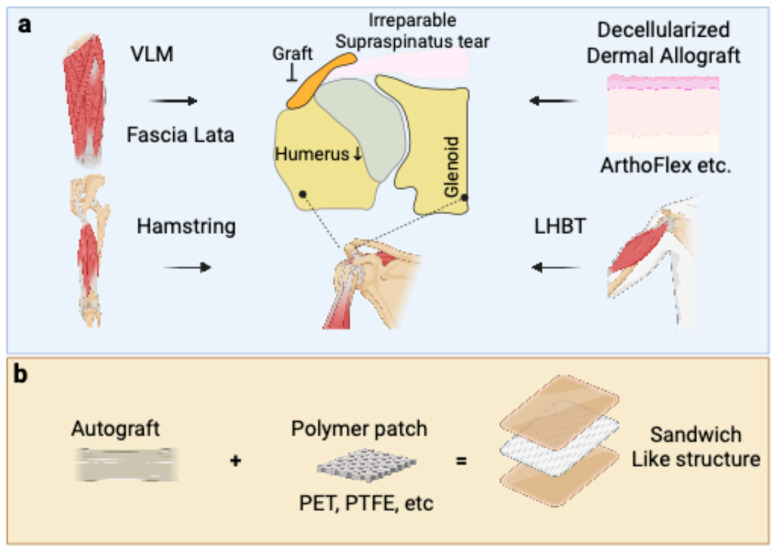
Schematic overview of current and emerging graft choices for the ASCR. (**a**) Current biological graft choices used to bridge the glenoid and the greater tuberosity of the humerus. These include autografts, such as fascia lata, hamstring tendon, and the long head of the biceps tendon (LHBT), as well as allografts like decellularized dermal tissue (e.g., ArthoFlex). (**b**) Emerging strategy involving a composite, sandwich-like structure that combines an autograft with a synthetic polymer patch made from materials such as polyethylene terephthalate (PET) or polytetrafluoroethylene (PTFE) to enhance the mechanical strength of the reconstruction.

The fascia lata autograft (FLA) harvested from the patient’s thigh was the graft material used in the original description of ASCR by Mihata et al. and remains a widely utilized option [17] (Figure 1).

FLA is a dense connective tissue rich in collagen. When used as an autograft, it brings with it a population of viable fibroblasts. These cells can contribute to matrix production and remodeling. Its autologous nature ensures perfect biocompatibility. The graft can be folded to achieve a desired thickness, typically 6–10 mm, which may enhance its mechanical strength and provide a more substantial scaffold for tissue integration.

The presence of viable cells and a native ECM scaffold promote robust biological incorporation. Studies have reported high healing rates with FLA. Mihata et al. documented a 91.7% healing rate at 1-year follow-up and 83.3% to 93% in other series. A 5-year follow-up study showed a 90% healing rate (27 out of 30 patients), indicating excellent durability. A systematic review indicated a pooled graft tear rate of only 9% for FLA autografts. This high healing potential is attributed to its optimal biological integration [17]. The primary drawback FLA is donor site morbidity, including pain, hematoma, scarring, or muscle herniation at the harvest site. The newly developed minimally invasive FLA harvesting technique leads to donor site satisfactory subjective results and good functional outcomes at 18 months after surgery [18]. A study of 66 patients found that FLA harvesting residual symptoms had minimal functional impact and were widely accepted by the patients [19].

### 3.2. Long Head of the Biceps Tendon (LHBT) Autograft

The LHBT, if available and of sufficient quality, can be used as an autograft for ASCR (Figure 1). As an autologous tendon, the LHBT contains viable tenocytes and an organized collagenous matrix. Its use may preserve some of its native vascular supply, potentially expediting the initial phases of healing [20]. The biological advantages are similar to other autografts, with the potential for good integration. Clinical outcomes have been reported as comparable to other graft types, with significant improvements in pain and function. Studies comparing LHBT autografts with an FLA found no significant differences in functional outcomes or relapse rates, suggesting a similar biological response [21,22,23].

### 3.3. Hamstring Tendon Autograft

Semitendinosus or gracilis tendons are other autologous tissues that can be used (Figure 1). These tendons also offer the benefits of viable cells and a native collagenous structure, promoting biological healing without an immunogenic response. Similar to other autografts, they are expected to integrate well. Their biological profile supports cellular repopulation and revascularization from host tissues [24].

## 4. Allografts: Balancing Availability with Biological Considerations

Allografts, sourced from human cadaveric donors, offer several advantages, including the avoidance of donor site morbidity, reduced operative time, and the availability of various tissue types and sizes. However, they introduce biological complexities related to processing, immunogenicity, and the absence of viable cells [25,26].

### 4.1. Acellular Dermal Allograft (ADA)

ADAs are a common allograft choice for ASCR. These grafts are processed to remove cellular components, theoretically reducing immunogenicity, while preserving the dermal collagen–elastin scaffold.

ADAs are essentially an acellular ECM scaffold. The processing aims to remove donor cells and antigens, but residual cellular material or antigens can sometimes elicit an inflammatory or immune response. The biological behavior of ADAs depends on the host’s ability to repopulate the scaffold with its own cells and revascularize it. The quality and thickness of the dermal matrix are crucial [27].

Healing of ADAs is a process of “creeping substitution,” where the host tissue gradually infiltrates and remodels the graft. This process is generally slower and potentially less robust than with autografts. Healing rates for dermal allografts are variable. Some studies report good outcomes, with one study showing a 77.5 ASES score and a 45% healing rate [28]. Another reported that significant differences were found in glenohumeral superior translation (ghST), subacromial peak contact pressure (sPCP), and cumulative deltoid force (cDF) when 3 mm thick grafts were replaced to 6 mm thick grafts, highlighting the importance of graft thickness for biological success [29]. Hirahara et al. reported a 25% graft tear rate (75% healing) [30]. A systematic review found a pooled graft tear rate of 7% for dermal allografts, which is comparable to tensor fascia lata (TFL) autografts in that specific analysis [31]. However, other studies have shown higher failure rates, with one study reporting a graft tear rate of 94.4% for xenografts and allografts combined, with the allograft healing rate specifically being only 7.7% in that cohort [32]. Re-tear rates for allografts in general have been reported to range from 19% to 70%. The variability likely reflects differences in processing, thickness, patient populations, and surgical technique. Thicker ADAs (>3 mm) have been associated with better outcomes [33], presumably because they provide a more substantial and durable scaffold for biological integration.

### 4.2. Fascia Lata Allograft

Similar to the autograft version, but sourced from a donor and processed. This graft provides a dense collagenous scaffold but lacks viable cells. Processing methods are critical to reduce immunogenicity [23]. Integration relies entirely on host cell migration and revascularization. One study reported a 40% complete healing rate at 6 month follow-up, suggesting a slower or less complete biological uptake compared to its autologous counterpart [34].

### 4.3. General Considerations for Allografts

The sterilization and decellularization processes, while necessary to reduce disease transmission and immunogenicity, can alter the biomechanical properties and biological responsiveness of the allograft ECM [35]. The absence of viable donor cells means that the entire burden of remodeling falls on the host’s cellular machinery, which can lead to a more protracted healing timeline and potentially a mechanically weaker construct during the early phases of incorporation.

## 5. Xenografts: Navigating Significant Biological Hurdles

Xenografts, derived from animal tissues (e.g., porcine dermis), offer potential advantages in terms of availability and cost. However, they present the most significant biological challenges related to immunogenicity.

### Porcine Dermal Xenograft

These grafts consist of an animal-derived collagen scaffold. Despite processing to remove cells and reduce antigenicity (e.g., alpha-gal epitopes), the risk of an adverse immunologic host response remains a major concern [36,37].

The biological response to xenografts can be characterized by significant inflammation and immune-mediated rejection. This can lead to poor graft integration, encapsulation, or even complete graft degradation and failure. Studies have reported procedural complication rates up to 30% with porcine dermal xenografts, with a substantial portion (e.g., 15%) due to immunologic graft rejection and another 15% due to graft failure from insufficient healing [38]. The high rate of adverse biological reactions often results in unacceptably high re-tear or failure rates.

## 6. Synthetic Grafts: Off-the-Shelf Scaffolds and Their Biological Interactions

Synthetic grafts, made from biocompatible polymers, offer an alternative by providing an off-the-shelf, sterile, and mechanically consistent option, eliminating concerns about donor site morbidity or disease transmission.

### 6.1. Non-Resorbable Synthetic Grafts (e.g., PTFE, PET–LARS)

Materials like polytetrafluoroethylene (PTFE) [39,40] or polyethylene terephthalate (PET) [41], such as the Ligament Augmentation and Reconstruction System (LARS), have been used (Figure 1b).

These materials are typically inert and non-resorbable. They act as a mechanical scaffold rather than undergoing true biological integration and remodeling into host-like tissue [42]. The host response usually involves encapsulation by a layer of fibrous tissue. There is no cellular repopulation or revascularization of the graft material itself [43].

While they can provide initial mechanical stability, the long-term biological interaction is one of tissue ingrowth around and into the interstices of the graft [44], rather than replacement of the graft. Okamura et al. reported improved clinical outcomes with a low graft rupture rate using synthetic grafts. The LARS ligament, when used to augment FLA, showed a significantly higher graft healing rate (91%) compared to polypropylene mesh augmentation (72%) [39]. This suggests that while the synthetic component provides structural support, the success in augmented repairs may still heavily rely on the biological activity of the accompanying autograft. Concerns with purely synthetic, non-resorbable grafts can include particulate wear debris over time and stress shielding of adjacent tissues.

### 6.2. Sandwich-Like Structure: Hybrid Grafts for Augmentation

**Figure 2:**
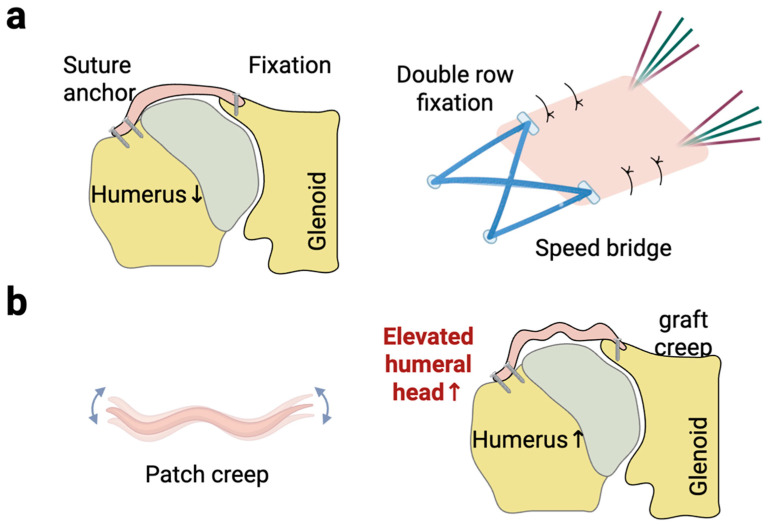
Schematic of autograft fixation of the ASCR and the mechanism of failure due to graft creep. (**a**) Graft fixation for ASCR. A graft is securely attached to the superior aspect of the glenoid and the greater tuberosity of the humerus using a “speed-bridge” configuration to ensure a broad and stable footprint. (**b**) Long-term failure mechanism known as “graft creep”, losing its initial tension and structural integrity. This elongation of the graft leads to a loss of its restraining function, resulting in the superior migration or elevation of the humeral head. The arrow indicates the direction of movement of the humeral head.

The hybrid or “sandwich-like” graft structure addresses the biomechanical limitations of an FLA alone, namely its variable thickness and potential for post-operative stretching or “patch creep” (Figure 2). This technique combines a synthetic scaffold for immediate mechanical stability with the superior histocompatibility of a surrounding biological graft for long-term integration. For instance, Polacek et al. developed a reinforced three-layer FLA using an internal non-resorbable suture mesh, which reliably achieved a desired graft thickness of ~7.6 mm, creating a stiff, standardized construct while preserving the autograft’s biological potential [45]. Similarly, Bi et al. created a three-layer graft by sandwiching a LARS ligament (PET) or polypropylene mesh within a folded FLA [41]. They reported a superior ASES score and UCLA score and a 91% healing rate with the LARS ligament, attributing this to its robust mechanical properties and biocompatibility that encouraged cellular ingrowth. This hybrid approach aims to engineer a graft that provides both the immediate stability necessary to restore glenohumeral kinematics and the biological viability required for durable healing. However, long-term clinical follow-up is needed in the future to reveal whether patients can benefit from this combination strategy.

## 7. The Role of Graft Thickness and Fixation in Supporting Biological Healing

Beyond the intrinsic biological nature of the graft material itself, other factors like graft thickness and the method of fixation play crucial roles in creating an optimal environment for biological healing.

### 7.1. Graft Thickness

A thicker graft generally provides a more substantial scaffold for cellular infiltration and revascularization. It may also offer greater initial mechanical strength, protecting the early healing processes from excessive strain. For instance, FLAs are often folded to 6–8 mm; this is supported by a biomechanical cadaveric study (n = 8) which found that an 8 mm thick FLA was superior in controlling superior humeral translation compared to a 4 mm graft when tested at multiple glenohumeral abduction angles (0°, 30°, and 60°) [46]. For dermal allografts, a thickness of >3 mm is associated with better pain relief, ASES scores, and healing rates, with grafts < 3 mm reported to fail to heal [29,47]. This suggests that a certain critical mass of scaffold material is necessary to support robust biological integration and withstand early mechanical stresses.

### 7.2. Fixation Methods

Stable fixation of the graft to both the glenoid and the greater tuberosity is paramount for successful biological healing. Micromotion at the graft–bone interface can disrupt the fragile early stages of revascularization and cellular ingrowth, leading to fibrous tissue formation instead of strong fibrovascular integration, and ultimately increasing the risk of re-tear [48].

Double-row suture anchor fixation (Figure 2) on the greater tuberosity is generally recommended as it creates a broader, more stable footprint for healing compared to single-row fixation [49,50]. Some studies have reported higher MRI graft failure rates (36.1%) and reoperation rates (36.1%) with single-row fixation. On the glenoid side, typically two or three suture anchors are used, with some evidence suggesting that three fixation points provide better stability [51,52,53,54]. Secure, multi-point fixation minimizes micromotion, allowing the biological processes of graft incorporation to proceed undisturbed, which is essential for all graft types, but particularly for acellular grafts that rely entirely on host tissue ingrowth.

## 8. Conclusions

The selection of graft material in Arthroscopic Superior Capsular Reconstruction is a critical decision that profoundly impacts surgical outcomes. This review underscores the significant influence of the graft’s inherent biological properties on its capacity to heal, integrate with host tissues, and resist re-tearing. All viable grafts choices for ASCR are summarized in Table 1.

Autografts, particularly FLAs, generally exhibit the most favorable biological profile due to their viability, non-immunogenicity, and native ECM, leading to high rates of healing and durable incorporation. Allografts, such as acellular dermal matrices, offer practical advantages but their acellular nature and the impact of processing necessitate a slower, host-driven integration process, resulting in more variable healing rates that are often dependent on factors like graft thickness. Xenografts, despite their availability, face substantial immunological barriers that frequently lead to rejection and failure. Synthetic grafts provide mechanical support as scaffolds, but their interaction with host tissue is typically one of encapsulation or ingrowth rather than true biological remodeling.

The biological characteristics of the chosen graft—its cellularity, ECM structure, immunogenicity, and potential for revascularization and remodeling—are inextricably linked to the success of ASCR. Furthermore, optimizing factors such as graft thickness and employing robust fixation techniques are essential to create a mechanical environment conducive to these biological healing processes.

Future research should continue to focus on developing or refining graft materials that enhance biological activity, perhaps through tissue engineering strategies, growth factor augmentation, or improved processing techniques for allografts. A deeper understanding of the interplay between graft biology, host response, and the biomechanical environment of the shoulder will further guide surgeons in making individualized graft choices to maximize healing potential and improve long-term outcomes for patients with irreparable rotator cuff tears. The quest for the ideal ASCR graft—one that combines optimal biological performance with ready availability and minimal morbidity—remains an important frontier in shoulder surgery.

## Figures and Tables

**Table 1:** Summary of grafts for Arthroscopic Superior Capsular Reconstruction (ASCR).

Graft Category	Specific Graft Type	Key Biological Properties and Healing Mechanism	Reported Clinical Outcomes (Healing/Re-tear Rates)	Advantages	Disadvantages and Challenges
Autografts	Fascia Lata Autograft (FLA)	Contains viable fibroblasts and a native collagen matrix; non-immunogenic. Heals via robust biological incorporation.	High healing rates reported: 91.7% at 1 year, 90% at 5 years. A systematic review found a pooled tear rate of only 9%.	Considered a gold standard; excellent biocompatibility and durability. fold to a desired thickness (6–10 mm)	Donor site morbidity (pain, scarring, muscle herniation at the harvest site). Potential for the graft to stretch over time (“graft creep”).
	Long Head of Biceps Tendon (LHBT)	Contains viable tenocytes and an organized collagen matrix. May preserve some native blood supply, aiding initial healing.	Functional outcomes and relapse rates are comparable to FLA.	Avoids a second incision site (e.g., the thigh). Uses available local tissue.	Only an option if the tendon is present and of sufficient quality and size.
	Hamstring Tendon	Contains viable cells and a native collagen structure. Promotes biological healing without an immune response.	Expected to integrate well, similar to other autografts. (Specific rates not provided in the text.)	Provides the inherent biological benefits of autologous tissue.	Donor site morbidity at the harvest location.
Allografts	Acellular Dermal Allograft (ADA)	Healing occurs via “creeping substitution”, a slow process of host cell repopulation.	Highly variable. Pooled tear rate of 7% was found in one review. Thicker grafts (>3 mm) perform better.	Readily available “off-the-shelf,” avoids donor site morbidity, and reduces operative time.	Slower, less robust healing compared to autografts. Risk of inflammatory response and highly variable outcomes.
	Fascia Lata Allograft	A dense, acellular collagen scaffold from a cadaveric donor. Integration relies completely on host cell migration.	Slower and less complete healing than its autograft counterpart. One study reported a 40% complete healing rate at 6 months.	Avoids donor site morbidity.	Lacks viable cells, leading to slower biological uptake.
Xenografts	Porcine Dermal Xenograft	An animal-derived collagen scaffold processed to remove cells and antigens.	Unacceptably high failure rates. Complication rates up to 30%, due to immunologic rejection (15%) or failure to heal (15%). One study showed a 64% tear rate at 2 years.	High availability and potentially lower cost.	Significant risk of a strong inflammatory and immune rejection response. Leads to poor integration, graft degradation, and frequent failure.
Synthetic Grafts	Non-resorbable (e.g., PTFE, PET–LARS)	Inert, biocompatible polymers that act as a mechanical scaffold. The host response is fibrous encapsulation or ingrowth, not biological remodeling.	Improved clinical outcomes with low graft rupture rates have been reported.	Provides immediate mechanical stability. Off-the-shelf, sterile, and mechanically consistent.	Lack of true biological integration. Long-term concerns include particulate wear debris and stress shielding of adjacent tissues.
	Sandwich-like Structure (Hybrid Grafts)	A composite graft combining a synthetic scaffold (for mechanical strength) with a biological graft, often an FLA.	A LARS ligament combined with FLA showed a 91% healing rate, superior to other augmentation. A hybrid of FLA and dermal allograft showed an 83.3% healing rate.	Aims to provide both immediate mechanical stability and long-term biological healing.	A newer technique that requires more long-term clinical follow-up to confirm its benefits.
